# Technostress, Perceived Organizational Support, and Burnout Among Healthcare Professionals: A Suppression Mediation Model

**DOI:** 10.3390/nursrep16070239

**Published:** 2026-07-10

**Authors:** Habib Alrashedi, Nader Alnomasy, Sudharani B. Banappagoudar, Ebtsam Abou Hashish

**Affiliations:** 1 Department of Medical Surgical Nursing, College of Nursing, University of Hail, Hail 2440, Saudi Arabia; n.alnomasy@uoh.edu.sa; 2 Department of Nursing, College of Applied Medical Sciences, King Faisal University, Al Ahsa 31982, Saudi Arabia; sbanappagoudar@kfu.edu.sa; 3 College of Nursing, King Saud bin Abdulaziz University for Health Sciences, Jeddah 21423, Saudi Arabia; abouhashishe@ksau-hs.edu.sa; 4 King Abdullah International Medical Research Center, Jeddah 22384, Saudi Arabia; 5 Ministry of National Guard Health Affairs, Jeddah 22384, Saudi Arabia; 6 Faculty of Nursing, Alexandria University, Alexandria 21526, Egypt

**Keywords:** technostress, burnout, perceived organizational support, path analysis, healthcare professionals, Saudi Arabia, digital health

## Abstract

**Background/Objectives:** This study aimed to assess technostress and burnout levels among healthcare practitioners, explore the relationships between technostress, perceived organizational support (POS), and burnout, and examine whether POS mediates this relationship using a suppression approach. The rapid proliferation of digital health technologies has increased technology-related job demands in healthcare settings. Technostress has become a major psychological risk factor associated with burnout, but evidence for its direct effects is inconsistent. Although organizational resources, especially perceived organizational support, might moderate this relationship, the underlying mechanism is not well understood, particularly in the context of rapidly digitalizing healthcare systems like Saudi Arabia’s. **Methods:** This was a cross-sectional, multicenter, descriptive-correlational study with a sample of 150 healthcare professionals conveniently selected from clinical settings in Saudi Arabia. Technostress Creators Scale, Copenhagen Burnout Inventory and Perceived Organizational Support Scale were used for data collection. Statistical analyses included Pearson correlations, multiple regression, bootstrap mediation analyses, and structural equation modeling. **Results:** Moderate levels of technostress were observed across all dimensions (M = 2.87–3.42). POS was positively related to technostress (r = +0.36, *p* < 0.001) and negatively related to burnout (r = −0.54, *p* < 0.001). The bivariate relationship between technostress and burnout was nonsignificant (r = −0.06, *p* = 0.437). Mediation analysis showed a significant negative indirect effect through POS (β = −0.19, *p* = 0.002) and a significant positive direct effect (β = +0.14, *p* = 0.019), indicating an inconsistent mediation (suppression effect). Structural equation modelling demonstrated excellent model fit consistent with a just-identified model. **Conclusions:** The findings suggest that the observed statistical associations are consistent with a suppression mediation model in which technostress was positively associated with burnout while also being associated with higher perceived organizational support, which in turn was negatively associated with burnout. POS plays a critical protective role but does not fully offset the harmful associations of technostress. Healthcare organizations should implement proactive support strategies, including continuous technical support, structured digital training, and active managerial engagement, to manage technostress and reduce burnout risk.

## 1. Introduction

The rapid spread of information and communication technologies (ICT) has fundamentally changed the provision of healthcare services globally. Frontline professionals, the primary users of these systems, must continually adapt to technological changes [1]. In this regard, Saudi Arabia’s Vision 2030 has resulted in greater investment in digital health infrastructure, such as electronic health records (EHRs), AI-driven decision support, telemedicine, and computerised clinical documentation systems [2,3]. While these digital innovations promise significant improvements in care quality, operational efficiency, and patient safety, their rapid implementation poses significant psychological risk for the healthcare workforce, especially in the form of technostress and its downstream effects, such as burnout [4,5,6].

Technostress is psychological strain that is experienced as a result of inability to deal with new computer technologies in a healthy manner. It was first conceptualised by Brod and later operationalised by Tarafdar et al. and Ragu-Nathan et al. [7,8,9]. The five dimensions of techno-overload, techno-invasion, techno-complexity, techno-insecurity and techno-uncertainty are collectively cognitively, emotionally and behaviourally burdensome for healthcare professionals who are expected to navigate evolving digital systems while maintaining high standards of patient care [4,10]. Although these technology-related demands are intuitively associated with burnout, the literature points to an inconsistent direct relation between these constructs, indicating the importance of intervening mechanisms that mediate this pathway [11,12].

Perceived organizational support (POS) refers to the extent to which employees feel that the organization values their contribution and cares about their well-being [13] and has been theoretically conceptualized as an important resource that can alter the stress–burnout process. Based on the Job Demands–Resources (JD-R) model [14], POS is a prototypical organisational resource that can buffer the negative impact of technological job demands on well-being. However, the exact mediating mechanism through which POS operates in the context of technostress and burnout in healthcare is empirically under-characterized. This issue is of particular salience in the Gulf regional context where burnout rates often soar above 40–60% [15,16].

Although previous studies have consistently linked technostress with burnout, most have concentrated on direct associations or simple buffering effects, without examining the underlying suppression mechanism through which organizational resources alter this relationship. In particular, whether perceived organizational support functions as a mediating resource that reshapes the observed association between technostress and burnout remains largely unexplored, especially among healthcare professionals working in rapidly digitalizing healthcare systems. Therefore, this study extends previous research by testing whether perceived organizational support functions as a suppression mediator that explains the inconsistent association between technostress and burnout. Specifically, the present study used path analysis and structural equation modelling to examine the direct, indirect, and total effects of technostress on burnout through the mediating pathway of POS among healthcare professionals in Saudi Arabia.

### 1.1. Conceptual and Theoretical Framework

The study is guided by two frameworks that provide explanations for the study variables.

The first framework is the Job Demands–Resources (JD-R) Model [14]. This model proposes that occupational well-being is shaped by a dual-process structure. In the energetic process, job demands—defined as physical, cognitive, or emotional requirements of work that necessitate sustained effort and are therefore associated with physiological and psychological costs—deplete employees’ resources and contribute to burnout. Within this framework, technostress constitutes a high-intensity job demand: the continuous pressure to adapt to new digital systems, maintain documentation accuracy in EHRs, respond to digital alerts, and update technical competencies all represent sustained cognitive loads that activate the health impairment process. In the motivational process, job resources—including organizational support, training opportunities, and managerial assistance—stimulate intrinsic motivation and engagement and buffer the negative consequences of demands. POS is a prototypical job resource within this framework: by communicating to employees that their efforts are valued and their well-being is protected, POS may provide access to psychological, informational, and organizational resources that could help offset resource depletion.

Accordingly, the JD-R model predicts that POS, as a job resource, should moderate and mediate the relationship between technostress (as a job demand) and burnout (as the outcome of the health impairment process). Recent JD-R work has demonstrated how organizational support dynamically buffers the psychological distress caused by after-hours digital demands, further supporting the positioning of POS as a resource in digitally intensive work contexts [17]. The motivational process, on the other hand, confirms that job resources such as organizational support buffer burnout by activating motivational processes. According to the JD-R model, both processes operate simultaneously, explaining why the relationship between demands and burnout may be altered by the availability of resources.

While the JD-R model delineates the pathways between demands, resources, and workplace outcomes, Conservation of Resources (COR) theory provides the underlying resource-based mechanism [18]. COR theory proposes that individuals strive to acquire, retain, and protect valued resources, and that psychological stress occurs when resources are lost, threatened, or fail to be replenished following significant investment. In the context of the present study, technostress creators function as active resource threats: cognitive overload depletes attentional reserves, techno-invasion erodes recovery time, techno-complexity generates self-efficacy loss, and techno-uncertainty produces anticipatory anxiety about professional obsolescence. These resource losses accumulate in a loss spiral that ultimately culminates in burnout.

Conversely, POS may function as a resource reservoir that helps offset resource depletion: when employees perceive that the organization values their contributions and invests in their well-being, they may gain access to supplementary emotional, informational, and instrumental resources that can counteract the depletion associated with technostress. This resource gain mechanism accounts for the protective and mediating function of POS in the present model. The two frameworks complement each other: the JD-R model specifies the dual-process pathways through which demands and resources produce burnout and engagement, while COR theory explains the directional mechanisms—resource loss and resource gain—that drive these processes.

#### 1.1.1. Technostress Creators

The five dimensions of technostress creators conceptualized by Tarafdar et al. and validated by Ragu-Nathan et al. are technology-related demands that collectively challenge the adaptive capacity of healthcare professionals [8,9]:

Techno-overload is the situation in which ICT-related expectations cause professionals to work faster, process more information, and work longer hours than is sustainable. In clinical settings, this translates into the need to manage electronic documentation, real-time data entry, and direct patient care simultaneously, presenting competing cognitive demands [8,19,20].

The concept of ‘techno-invasion’ refers to the invisible boundaries between work and private life, made feasible by ICTs that enable continuous communication and availability. The omnipresence of mobile alerts, the potential of telehealth care, and the requirement of electronic communication even after hours promote a ubiquitous feeling of “always on” that takes away from chances for recovery thought to be necessary for psychological repair [5,6].

Techno-complexity is the psychological cost of the seeming complexity of comprehending and using complex digital systems. Complexity in healthcare creates inadequacy and self-doubt that undermine professional confidence [4,10]. This is particularly true for systems that require high digital competence, such as clinical decision support systems, EHRs, and telehealth systems. Techno-insecurity is the relationship between worry and perceived job security risks. Techno-insecurity can threaten job security through automation, artificial intelligence, and the expectation that workers will continuously upskill technologically. Professionals who feel that they do not have the ability to accommodate the technology demands are prone to anxieties of being replaced [11,21].

Techno-uncertainty is the mental strain produced by continual system updates, version upgrades and the insecurity of the digital infrastructure. These common difficulties need a re-learning of traditional workflows and a recalibration of operational competencies of professionals. This dimension has been one of the most prominent technostress generators in nursing and healthcare research [6,21,22].

#### 1.1.2. Perceived Organizational Support

Perceived Organizational Support (POS) is employees’ global opinions about how much the organization values their contribution and cares about their well-being, according to Eisenberger et al. [13] and based on Social Exchange Theory by Blau [23]. The present investigation has used the POS Scale [13]. The POS Scale has multiple related POS dimensions:

The organizational valuation of contributions is connected to the employees’ impression of recognition and appreciation of their work, talents and professional judgment by the organization. This view is especially significant in high demand digital transformation environments where professional work is frequently redirected to system mastery rather than direct treatment [24].

Care for employee well-being conveys the idea that the organization cares about the physical and psychological health of employees, even during periods of technological intensification. This is promoted by organisations showing tangible concern in the form of workload monitoring, screening for burnout and support interventions [25].

Recognition and fairness are linked to equitable distribution of resources, access to training, and workload distribution which are notably important when digital transformation leads to disparity in technological proficiency and access across units and shifts [26].

Access to resources and managerial support means providing technical assistance, time for training, helpful IT helpdesks and involved supervisors to assist professionals in handling technology demands [24].

Burnout, a multidimensional syndrome of sustained psychological exhaustion, is defined with the Copenhagen Burnout Inventory [27] across three distinct but interrelated domains:

Personal burnout is a state of deep physical and psychological exhaustion that extends outside the work role and affects the functioning and quality of life of the individual. It reflects the extent of tiredness, energy depletion and emotional exhaustion experienced by professionals regardless of the causes related to their work [27,28]. The highest level of individual burnout was in line with regional evidence that has documented the extensive psychological burden of technology-intensive clinical work [16].

Work-related burnout is a type of burnout that is specifically linked to occupational stressors such as the physical and emotional exhaustion of workload, role ambiguity and the continuous cognitive demand of digital documentation and navigating systems [28,29].

Client-related burnout is defined as emotional exhaustion from working with patients or clients, encompassing compassion fatigue, emotional depletion from therapeutic interactions, and the feeling of being unable to provide adequate care when cognitive resources are consumed by technology demands, a dimension with direct implications for quality of care and patient safety [28,29].

### 1.2. Research Hypotheses

Based on the JD-R model [14] and COR theory [18], the following directional hypotheses were formulated:

**H1.**  *The factors leading to technostress will be positively and significantly related with burnout among healthcare professionals.*

**H2.**  *There will be a significant association between technostress creators and perceived organizational support (POS) among healthcare professionals.*

**H3.**  *Perceived organizational support (POS) is negatively and significantly associated with the burnout of healthcare professionals.*

**H4.**  *Perceived organizational support mediates the relationship between technostress creators and burnout through a suppressed mediation, such that the indirect effect through POS is statistically significant and occurs concurrently with a statistically significant direct effect, resulting in an almost zero total effect.*

**Figure 1:**
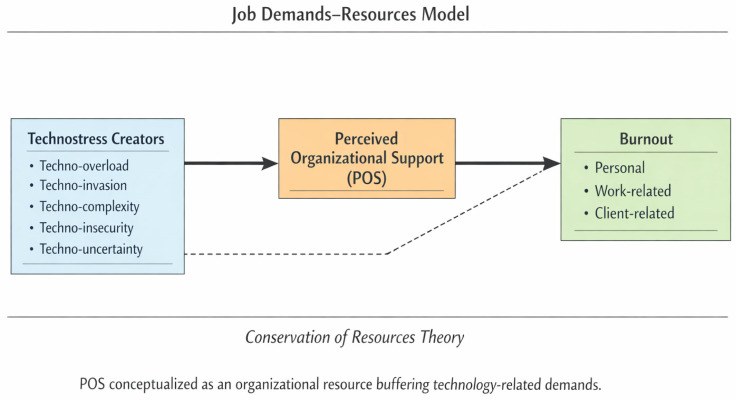
Conceptual Framework.

See Figure 1 for the proposed conceptual framework.

### 1.3. Significance of the Study

Saudi Arabia’s Vision 2030 is driving an acceleration in the digitalization of healthcare, and understanding the pathways through which technological demands translate into burnout in the workforce is both a scientific and practical imperative. Current regional studies have concentrated primarily on burnout as a consequence of staffing shortages and workload, with little regard for the psychological burden specific to technology [15,16]. This study offers evidence-based insights to guide organizational strategies in line with Vision 2030 and international best practices in digital workforce sustainability through rigorous path analytic and SEM approaches to unpack the mechanisms of burnout in digitally intensive contexts.

### 1.4. Aim of the Study

The aim of this study was to: (1) evaluate the levels of technostress, POS, and burnout among health care professionals; (2) investigate the direct relationships between technostress, POS, and burnout; and (3) test whether POS mediates the relationship between technostress and burnout.

## 2. Materials and Methods

### 2.1. Study Design and Setting

This was a cross-sectional, descriptive-correlational study in clinical settings of Saudi Arabia. The study was designed and reported according to the STROBE (Strengthening the Reporting of Observational Studies in Epidemiology) guidelines for cross-sectional studies. The setting was healthcare facilities with advanced digital health infrastructure, where nursing and clinical staff routinely interacted with Electronic Health Records (EHRs), Computerized Physician Order Entry (CPOE), digital medication administration records (eMAR) and telehealth platforms.

### 2.2. Sampling and Participants

A convenience sample of 150 healthcare professionals was recruited from three secondary- and tertiary-level hospitals in the Riyadh and Al Qassim regions of Saudi Arabia between September and December 2024. Eligible participants were healthcare professionals with at least one year of experience who routinely interacted with clinical information technology systems (EHRs, CPOE, or telehealth platforms). Students, interns, and non-clinical administrative staff were excluded. Recruitment was conducted through institutional email invitations and QR code-linked surveys displayed at clinical stations, supplemented by paper-based questionnaires distributed to professionals who did not have regular email access. All ward and unit charge nurses and department supervisors were contacted to facilitate purposive distribution within eligible clinical units. Sample size was determined using G*Power 3.1 [30]. Assuming a medium effect size (f^2^ = 0.15), α = 0.05, statistical power = 0.80, and two predictors in the regression model, the minimum required sample size was 107 participants. The final sample of 150 exceeded this threshold, providing adequate power for stable path estimation in single-indicator path models [31]. Of 187 distributed questionnaires, 150 were completed and met inclusion criteria, yielding a response rate of 80.2%.

### 2.3. Data Collection Instruments

#### 2.3.1. Technostress Creators Scale

Technostress was measured by using the Technostress Creators Scale developed by Tarafdar et al. [8]. The scale includes 23 items grouped in five validated dimensions: techno-overload (5 items), techno-invasion (4 items), techno-complexity (5 items), techno-insecurity (5 items), and techno-uncertainty (4 items). All items are assessed on a 5-point Likert scale (1 = strongly disagree to 5 = strongly agree), with higher scores indicating higher perceived technostress. The scale had good psychometric properties in several international and regional healthcare studies [9]. In the present study, reliability coefficients ranged from α = 0.81 to 0.89.

#### 2.3.2. Copenhagen Burnout Inventory (CBI)

Burnout was measured using the Copenhagen Burnout Inventory [27], a 19-item multidimensional instrument measuring burnout across three domains: personal burnout (10 items), work-related burnout (3 items), and client-related burnout (6 items). Items are rated on a 5-point Likert scale ranging from 0 (never/almost never) to 100 (always), with higher scores indicating higher levels of burnout. The CBI has been extensively validated in nursing and healthcare workforce research internationally and in the Middle Eastern context [16,29]. Reliability in the overall scale was α = 0.84 and the subscale reliabilities ranged from 0.79 to 0.88 in the present study.

#### 2.3.3. Perceived Organizational Support Scale (POS)

Perceived organizational support was measured with the 8-item POS Scale developed by Eisenberger et al. [13] that assesses the extent to which employees perceive that the organization values their contributions and cares about their well-being. Items were rated on a 7-point Likert scale (1 = strongly disagree, 7 = strongly agree). The scale has shown high internal consistency (Cronbach’s α = 0.90) and good construct validity in different occupational settings [13,24]. The scale showed good reliability in the present study (α = 0.92).

### 2.4. Validity and Reliability

All three measures were administered in their original validated English versions. The use of English-language instruments was appropriate for this sample because: (1) English is the official language of clinical documentation and inter-professional communication in the participating Saudi hospitals; (2) all eligible participants held at minimum a bachelor-level healthcare qualification, for which English proficiency is a national prerequisite in Saudi accredited programmes; and (3) validated Arabic translations of the Technostress Creators Scale and the 8-item POS Scale with adequate psychometric properties were not available in the peer-reviewed literature at the time of the study. Content validity was assessed via expert review by five specialists in nursing informatics and psychometrics, yielding a content validity index (CVI) ≥ 0.80 for all items. Exploratory factor analysis of the technostress scale in the present sample confirmed the five-factor structure [8], with factor loadings ranging from 0.62 to 0.84, KMO = 0.81, and Bartlett’s test significant (χ^2^ = 1842.3, *p* < 0.001), supporting construct validity. Cronbach’s alpha coefficients assessed internal consistency and ranged from 0.79 to 0.92 for all subscales and composite scales, exceeding the recommended threshold of α ≥ 0.70 [32]. A pilot study with 5% of the target sample confirmed the clarity and comprehensibility of the instruments, with no modifications needed.

### 2.5. Data Collection

Ethical approval was obtained prior to distribution of standardised questionnaires, which were distributed using a mixed-mode approach; approximately 60% electronically (online survey via email and QR codes at clinical stations) and 40% in paper format. Individual healthcare professionals were contacted through institutional e-mail and secure messaging platforms. Data collection was preceded by obtaining informed consent. Data were collected over a period of four months.

### 2.6. Data Analysis

Data were analysed using SPSS version 25 with descriptive statistics, reliability, Pearson correlations and regression and with SEM using semopy. Of the 187 returned questionnaires 150 met inclusion criteria. Normality was checked using Shapiro–Wilk test. To test for common method bias, Harman’s single factor test was conducted. The first unrotated factor accounted for 28.3% of the total variance which was below the 50% cutoff point, suggesting that common method bias is unlikely to pose a threat to the findings. H4 was tested using bootstrap-based mediation analysis (5000 iterations) with bias-corrected 95% CIs. For the evaluation of SEM fit, CFI (≥0.95), GFI (≥0.95), AGFI (≥0.90), NFI (≥0.95), TLI (≥0.95), and RMSEA (≤0.06) were analyzed. Multicollinearity was tested by VIF. Statistical significance was *p* ≤ 0.05.

Descriptive statistics characterized the sample and variable distributions. Pearson correlations explored bivariate relationships among burnout, POS, and technostress. Multiple regression identified predictors of POS and burnout. Bootstrap mediation analysis (5000 samples, bias-corrected 95% CIs) tested H4. SEM fit was evaluated using CFI, GFI, AGFI, NFI, TLI (thresholds stated above), and RMSEA. Statistical significance was set at *p* ≤ 0.05.

### 2.7. Ethical Considerations

The study was performed in adherence to the ethical principles of the Declaration of Helsinki. The Institutional Review Board (IRB) approved data collection prior to its initiation. Informed written consent was obtained from all participants, and they were informed of the voluntary nature of participation, the right to withdraw without consequence and the confidentiality and anonymity of their data. All research materials were anonymized, and data were securely stored with restricted access throughout the study.

## 3. Results

### 3.1. Demographic and Work Characteristics

Table 1 presents a summary of demographic and professional characteristics (N = 150). The sample comprised nurses (n = 78, 52.0%), physicians (n = 31, 20.7%), pharmacists (n = 19, 12.7%), and allied health professionals including radiographers, laboratory technicians, and respiratory therapists (n = 22, 14.7%). Most participants were men (59.3%). Almost half held a bachelor’s degree (48.0%), and 32.7% held a master’s degree. The largest experience cohort comprised professionals with 6–10 years of service (32.0%), and most had 4–7 years of organizational tenure (34.7%), indicating a predominantly mid-career workforce with substantial exposure to ongoing digital transformation.

### 3.2. Reliability and Descriptive Statistics

**Figure 2:**
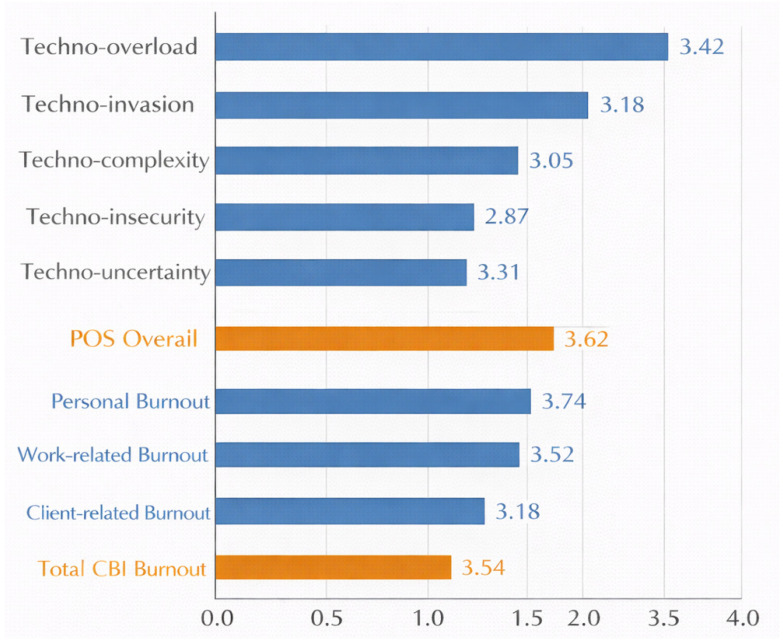
Mean Score for Study Variables.

Table 2 presents reliability coefficients, means, and standard deviations for all constructs in the study. The dimensions of technostress had acceptable to good internal consistency (α = 0.81 (techno-invasion) to α = 0.89 (techno-overload)). The mean scores of technostress dimensions ranged from 2.87 (techno-insecurity) to 3.42 (techno-overload) with techno-overload and techno-uncertainty being the most prominently perceived stressors. The POS showed good reliability (α = 0.92, M = 3.62, SD = 0.74) and indicates that participants perceived a moderate-to-good level of organizational support. Overall, burnout measured by the CBI was good (α = 0.84, M = 3.54, SD = 0.76). The most elevated burnout dimension was personal burnout (M = 3.74). The average score of the variables studied is presented in Figure 2.

### 3.3. Correlation Analysis (Hypotheses 1, 2 and 3)

Pearson correlation analysis was conducted to test the hypothesised bivariate relationships between technostress, POS and burnout (Table 3). However, the results did not support H1 in its simple bivariate form as technostress and burnout did not present a significant direct correlation (r = −0.057, *p* = 0.437) whereas technostress was significantly positively correlated with POS (r = +0.357, *p* < 0.001), supporting H2. In line with H3, POS was significantly and moderately to strongly negatively correlated with burnout (r = −0.537, *p* < 0.001).

### 3.4. Regression Analyses

Table 4 shows the four regression models with unstandardised (B) and standardised (β) coefficients. Consistent with the correlation results, Model 1 showed no significant bivariate association between technostress and burnout, whereas Model 2 showed that technostress significantly predicted POS and Model 3 showed that POS significantly predicted lower burnout. Importantly, in Model 4 (multiple regression), technostress was a significant positive predictor of burnout (B = +0.14, β = +0.16, *p* = 0.019), after controlling for POS. POS continued to exert its negative influence (B = −0.47, β = −0.59, *p* < 0.001) and the two predictors explained 30% of the variance in burnout. No multicollinearity problems were detected (VIF < 1.15).

### 3.5. Mediation Analysis (Hypothesis 4)

Table 5 provides the detailed results of the bootstrap mediation analysis. The indirect effect of technostress on burnout via POS was significant and negative (β indirect = −0.19, 95% CI [−0.30, −0.08], *p* = 0.002), supporting H4. The non-significant total effect, together with the significant positive direct effect and significant negative indirect effect, indicated inconsistent mediation or a suppression effect. Thus, POS revealed opposing pathways that were not visible in the simple bivariate association.

### 3.6. Structural Equation Modelling

**Figure 3:**
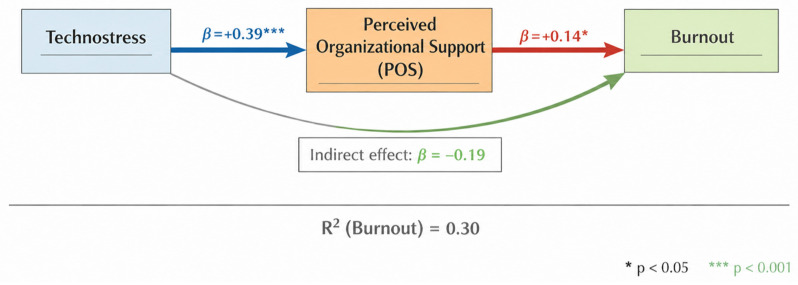
Structural Path Model.

SEM results were consistent with the regression and mediation findings. As shown in Figure 3, the direction and significance of the structural paths were consistent with the regression and mediation analyses. The model had almost perfect global fit indices (CFI = 1.011; GFI = 1.000; AGFI = 1.000; NFI = 1.000; TLI = 1.043; RMSEA = 0.000; χ^2^ ≈ 0.000, *p* > 0.05). These values are consistent with the just-identified (saturated) three-variable path model, where degrees of freedom are zero and global fit statistics are mathematically not informative of model-data correspondence beyond path significance. Therefore, global fit indices should be interpreted with caution. Substantive validation rests on the significance and direction of individual path coefficients. TS and POS accounted for 30% of the variance in burnout (R^2^ = 0.30).

## 4. Discussion

The Job Demands–Resources [14] and Conservation of Resources theories [18] provide the guiding frameworks for interpreting the direct and indirect pathways through which technostress was associated with burnout among healthcare professionals, with perceived organizational support serving as the theoretically grounded mediating variable. The findings contribute additional empirical insights, particularly the identification of an inconsistent mediation (suppression effect) that advances understanding of how digital transformation may affect the psychological well-being of healthcare workers.

### 4.1. Descriptive Levels of Technostress, POS, and Burnout

Moderate levels of technostress were observed across all five dimensions, with techno-overload and techno-uncertainty emerging as the most salient perceived stressors. This finding is consistent with several international and regional studies documenting the dual burden of volume and unpredictability as the predominant sources of technology-related distress in health care. Golz et al. [4] reported the increasing concern of technostress in nursing globally, with overload and uncertainty as the dominant dimensions. These findings were aligned with Wirth et al. [6], who found significant associations between techno-overload and burnout indicators. Kräft et al. [5] also directly linked digital stress intensity to emotional exhaustion and work–privacy conflict, thus underlining the energetic depletion route of the JD-R model. In the Gulf context, Keshavarz et al. [21] found that 41% of healthcare professionals experienced moderate technostress and techno-uncertainty was the main contributor.

The dominance of “techno-overload” reflects the well-documented rise in documentation and administrative tasks that come with the adoption of EHRs [19,20]. Simultaneously, the salience of techno-uncertainty, driven by constant system updates and evolving digital workflows, is consistent with findings in nursing contexts reported by Brandon and Keshavarz et al. [21,22]. These two dimensions are different but converging routes to depleting psychological resources: overload depletes cognitive capacity via volumetric demands, and uncertainty depletes cognitive capacity via anticipatory anxiety and re-learning requirements.

POS was moderate. This level is sufficient to buffer the immediate technology demands but shows significant scope for improvement in provisioning institutional support. Moderate POS may partly transform techno-distress into more manageable experiences, without fully removing the resource depletion pathway, in line with Barello et al. who showed that moderate organizational support buffered emotional exhaustion during COVID-19 without fully removing burnout risk [25].

All three CBI dimensions revealed moderate to high levels of burnout, with personal burnout scores being higher than work-related and client-related burnout. This pattern is in line with regional evidence indicating a considerable burden of burnout among Middle Eastern healthcare workers, often exceeding 40–60% [15,16]. The relatively high personal burnout dimension indicates that technology-intensive demands are not confined to the occupational role functioning, but are relevant to the overall vitality of professionals, in line with COR theory’s prediction of pervasive resource depletion consequences [18].

### 4.2. Inconsistent Mediation (Suppression Effect): Opposing Pathways and Their Clinical Significance

A key finding of this study is the empirical evidence of inconsistent mediation (suppression effect) in the relationship between technostress and burnout. The bivariate analysis revealed a non-significant correlation between technostress and burnout, which might suggest no meaningful association. However, path analysis and SEM revealed simultaneously significant positive direct and significant negative indirect effects through POS. The magnitude of these opposing effects produced a near-zero total effect that masked both underlying pathways. This pattern of suppression illustrates that bivariate correlations may be misleading in complex multivariate occupational health systems, where opposing indirect and direct effects cancel each other out [33].

The positive indirect pathway, whereby technostress was associated with higher POS which in turn was associated with lower burnout, is consistent with the JD-R buffering hypothesis [14] and COR resource caravan mechanisms [17,18]. One plausible interpretation is that, as technology-related demands become more visible, organizations may respond by strengthening supportive practices, including accelerated training programmes, dedicated IT helpdesks, supervisory check-ins, and peer mentoring, which replenish professionals’ depleted reserves [25]. Because the present study employed a cross-sectional design, however, this interpretation remains hypothetical and cannot confirm that such a mechanism actually operates in practice. Directionality requires confirmation through longitudinal research.

The significant positive direct association between technostress and burnout, visible only after statistically controlling for POS, is consistent with the theoretically expected harmful pathway. This finding aligns with Consiglio et al., who found that technostress creators were associated with burnout and poorer psychological health among remote workers even when resources were available [11]. Shaban et al. similarly documented that technostress contributes to burnout among critical care nurses, with emotional intelligence partially mediating this relationship [10]. Wirth et al. found that technical support offers moderated—but did not eliminate—the association between technostress indicators and burnout in German hospital nurses, consistent with the present finding that POS partially but not completely cancels the direct harmful pathway [6].

### 4.3. Perceived Organizational Support as a Mediating Resource

POS was a strong negative predictor of burnout, accounting for approximately 29% of the burnout variance in simple regression and retaining significance in the full model. These findings are consistent with the organizational support theory of Eisenberger et al. and with the broader empirical literature linking POS with reduced burnout in healthcare contexts [13]. Adan described organizational support—through recognition, resource availability, and leadership engagement—as one of the strongest protective factors against nurse burnout [24]. Barello et al. found that perceived organizational support was associated with significantly lower emotional exhaustion in healthcare professionals during the COVID-19 pandemic [25].

The positive association between technostress and POS is the most theoretically distinctive and clinically relevant finding of this study, and it warrants careful, dedicated interpretation. One possible explanation is that organizations experiencing higher levels of digital demands may respond by providing greater technical assistance, managerial engagement, and training opportunities, resulting in higher perceived organizational support among employees facing the greatest technostress burden. This reactive organizational dynamic is plausible within the JD-R 3.0 framework, in which Li et al. demonstrated that organizational support responds dynamically to digitally induced employee distress [17]. An alternative and equally plausible interpretation is that organizations with stronger pre-existing support cultures simultaneously invest more in digital infrastructure and training, so that both technostress and POS appear elevated without a causal relationship between them.

### 4.4. Clinical Translation: What Does Suppression Mean in Real Hospitals?

The suppression mechanism has an important, counter-intuitive clinical implication. The near-zero bivariate association between technostress and burnout in digitally intensive hospital settings may lead administrators to conclude that digital demands do not meaningfully affect burnout. The path model reveals a significant positive direct path (TS → Burnout) that is currently being masked by a strong protective indirect path (TS → POS → Burnout). Where organizations withdraw or reduce POS—by cutting IT support budgets, reducing training frequency or disengaging managerial oversight—the protective pathway is weakened and the suppressed harmful pathway surfaces as rapidly rising burnout. This is a plausible clinical implication that requires confirmation through longitudinal evidence. Healthcare leaders need to recognize that the apparent absence of a technostress–burnout problem may itself be indicative of successful, and potentially fragile, POS-mediated suppression that needs to be actively and continuously maintained [6,34].

### 4.5. Theoretical Interpretation

The overall pattern of findings is coherently interpretable within the JD-R model and COR theory. The positive direct association between technostress and burnout—visible only after controlling for POS—is consistent with the JD-R energetic process, in which demands deplete psychological resources and contribute to burnout. POS exerted a strong negative association with burnout, consistent with the JD-R motivational process through which resources buffer the health impairment pathway. The suppression mediation architecture reflects these two processes operating simultaneously and in opposite directions, producing the near-zero bivariate effect observed between technostress and burnout. From the COR perspective, the findings may suggest a dynamic resource caravan process: technostress threatens resource depletion, and the positive association between technostress and POS may reflect a compensatory resource mobilization process, whereby organizations or individuals seek to offset losses through additional support; this chain then reduces burnout through the indirect pathway, though it cannot fully neutralize the direct depletion effect [17,18]. Because this interpretation is based on cross-sectional data, causal conclusions cannot be drawn.

Tarafdar et al. differentiated techno-distress, arising when demands exceed resources, from techno-eustress, when demands are perceived as manageable challenges [34]. The present findings suggest that access to POS may help professionals shift from techno-distress toward a more manageable experience, not by reducing the stressor but by replenishing sufficient resources to sustain coping.

Accordingly, the present findings should be interpreted as statistical evidence consistent with several theoretical explanations rather than confirmation that organizations systematically increase support in response to technostress.

### 4.6. Strengths and Limitations

This study’s theoretical foundation in the JD-R model and COR theory is one of its main strengths. Another strength is the application of rigorous statistical methods, including bootstrap-based mediation analysis and SEM, to examine the complex relational architecture between technostress, POS, and burnout. The use of three well-validated instruments with good psychometric properties across diverse healthcare and cultural settings enhances the credibility of the findings. The identification of the suppression mediation pattern provides additional empirical evidence on the mechanisms through which technostress relates to burnout in digitally intensive healthcare contexts.

Several limitations must be recognized. First, the cross-sectional design precludes causal inference; the directionality of all identified pathways is theoretically grounded but must be confirmed with longitudinal data incorporating multiple measurement points. Second, all data were self-reported, introducing the potential for common method bias. To evaluate this risk, Harman’s single-factor test was conducted; the first unrotated factor accounted for 28.3% of total variance, below the 50% threshold, suggesting that common method bias is unlikely to substantially distort the findings. Third, the convenience sample of 150 professionals drawn from three hospitals in two Saudi regions limits external validity. The use of convenience sampling from three hospitals may further limit transferability to healthcare professionals working in different organizational structures, staffing models, and healthcare systems. Findings should be generalized cautiously to other professional categories and cultural contexts; future research should employ probability-based sampling across multiple sites and health system types.

Fourth, subgroup analyses by profession, gender, years of experience, or educational level were not conducted because they were beyond the predefined study objectives and the available sample size would have provided insufficient statistical power for multiple simultaneous comparisons; future studies with larger samples should examine whether the suppression mechanism varies across professional groups. Fifth, the instruments were administered in English; while English is the language of clinical documentation in the participating institutions and all participants met minimum English-proficiency requirements for their qualifications, responses from professionals for whom English is not a primary language may carry some measurement imprecision. Sixth, the model explained approximately 30% of the variance in burnout (R^2^ = 0.30), indicating that approximately 70% of burnout variance remains unexplained. Additional variables not included in the present model—such as workload, staffing adequacy, resilience, leadership style, organizational culture, digital competence, and individual coping strategies—may contribute substantially to burnout and should be incorporated in future studies [14,18].

## 5. Conclusions

The present study provides empirical evidence on the inconsistent mediation (suppression effect) of perceived organizational support in the technostress–burnout relationship among healthcare professionals in the rapidly digitalizing context of Saudi Arabia. The findings suggest that technostress was directly associated with higher burnout, but was also associated with higher perceived organizational support, which in turn was associated with lower burnout through an indirect protective pathway. This suppression pattern may explain why the bivariate association between technostress and burnout was negligible, as the opposing direct and indirect effects masked each other. Techno-overload and techno-uncertainty emerged as the most prominent stressor dimensions, and personal burnout was the most elevated outcome.

SEM path estimates confirmed the proposed model structure; global fit indices reflect the just-identified model and should be interpreted at the level of individual path coefficients. These findings underscore the importance of proactive organizational investment in comprehensive support infrastructures—including technical assistance, digital literacy programmes, supervisory engagement, and psychological safety practices—as strategic investments in workforce resilience during accelerating digital transformation. The protective association of POS with burnout is substantial but does not fully offset the direct associations of technostress, underscoring the importance of sustained and comprehensive support provision.

## 6. Practical Implications for Practice, Management, Policy, and Future Research

The findings emphasize the importance of accessible, workflow-integrated digital support systems for clinical practice. Plausible interventions targeting techno-overload and techno-uncertainty may include simplified EHR interfaces, real-time IT helpdesks, and digitally aligned workflow design. Peer mentoring programmes and unit-based digital champions may also serve as frontline resources for professionals experiencing techno-complexity and techno-insecurity [6,24].

With respect to healthcare and nursing management, the observed association between technostress and POS—whereby professionals experiencing greater technology-related demands were also more likely to report higher organizational support—suggests that proactive rather than reactive support provisioning may be beneficial. Healthcare managers may consider monitoring technostress as an early indicator of burnout risk and strengthening support infrastructure during digital implementation phases, system update cycles, or periods of high demand intensity. The visibility of managerial engagement and explicit recognition of technological burdens may represent practical levers for maintaining perceived organizational support, as supported by prior literature [6,25].

At the policy level, technostress monitoring could be integrated into routine workforce health assessments within digital health initiatives consistent with Vision 2030. Policies that formalize IT training standards and technical support response times, and that include clinical staff in the design of digital systems, may represent targeted and plausible approaches to sustaining workforce well-being during accelerating digitalization [2].

Future studies should employ longitudinal and mixed-method designs to capture the temporal dynamics of the technostress–POS–burnout pathway and to examine whether the suppression pattern is sustained or attenuated as digital transformation matures. Moderating variables—such as resilience, digital self-efficacy, leadership style, and professional identity—may further illuminate the conditions under which POS is most effective at buffering burnout. Multi-site, cross-cultural studies are necessary to evaluate the generalizability of the suppression mediation model. Future research should also include objective technology use metrics along with self-report measures to provide a more complete picture of digital demand–burnout mechanisms.

## Figures and Tables

**Table 1:** Sample Demographic Characteristics (N = 150).

Variable	n	%
Gender		
Male	89	59.3
Female	61	40.7
Education Level		
High School/Diploma	18	12.0
Bachelor’s Degree	72	48.0
Master’s Degree	49	32.7
Doctoral Degree	11	7.3
Years of Work Experience		
<2 years	22	14.7
2–5 years	41	27.3
6–10 years	48	32.0
>10 years	39	26.0
Years in Current Organization		
<1 year	19	12.7
1–3 years	38	25.3
4–7 years	52	34.7
>7 years	41	27.3

**Table 2:** Reliability Coefficients and Descriptive Statistics for Study Constructs.

Construct/Subscale	α	M	SD
Technostress Creators			
Techno-overload (5 items)	0.89	3.42	0.78
Techno-invasion (4 items)	0.81	3.18	0.82
Techno-complexity (5 items)	0.84	3.05	0.91
Techno-insecurity (5 items)	0.84	2.87	0.86
Techno-uncertainty (5 items)	0.82	3.31	0.79
Perceived Organizational Support—POS			
POS Overall (8 items)	0.92	3.62	0.74
Burnout—Copenhagen Burnout Inventory, CBI			
Personal Burnout (10 items)	0.88	3.74	0.83
Work-related Burnout (3 items)	0.79	3.52	0.91
Client-related Burnout (6 items)	0.83	3.18	0.87
Total CBI Burnout (19 items)	0.84	3.54	0.76

Note. α = Cronbach’s alpha. M = mean composite score. SD = standard deviation. All values exceed α ≥ 0.70.

**Table 3:** Pearson Correlation Analysis Among Technostress, POS, and Burnout.

Variable Pair	Pearson r	*p*-Value	Interpretation
Technostress ↔ POS	+0.36	<0.001	Moderate positive, significant
Technostress ↔ Burnout	−0.06	0.437	Negligible, not significant
POS ↔ Burnout	−0.54	<0.001	Moderate-strong negative, significant

Note. POS = perceived organizational support; N = 150.

**Table 4:** Summary of Linear Regression Models (Unstandardised B and Standardised β Coefficients).

Model	Predictor(s)	B	SE	β	R^2^	*p*
Model 1: Technostress → Burnout (Direct, Simple Regression)						
1	Technostress (TS)	−0.05	0.06	−0.06	0.00	0.437
Model 2: Technostress → POS (Simple Regression)						
2	Technostress (TS)	+0.39	0.07	+0.36	0.13	<0.001
Model 3: POS → Burnout (Simple Regression)						
3	POS	−0.43	0.05	−0.54	0.29	<0.001
Model 4: TS + POS → Burnout (Multiple Regression)						
4a	Technostress (TS)	+0.14	0.06	+0.16	0.30	0.019
4b	POS	−0.47	0.05	−0.59	0.30	<0.001

Note. TS = technostress total composite; B = unstandardised coefficient; β = standardised coefficient; SE B = standard error of B. Models 4a and 4b report coefficients for each predictor in the multiple regression model. R^2^ reported for the overall Model 4. N = 150.

**Table 5:** Bootstrap Mediation Analysis: Indirect Effect of Technostress on Burnout via POS (5000 Bootstrap Samples).

Path	Coeff. (B)	SE	t	*p*-Value	95% CI LL	95% CI UL
Direct Paths						
a path: TS → POS	+0.39	0.08	+5.23	<0.001	+0.24	+0.55
b path: POS → Burnout	−0.43	0.05	−8.73	<0.001	−0.53	−0.33
Direct: TS → Burnout	+0.14	0.06	+2.39	0.019	+0.02	+0.26
Total and Indirect Effects (Bootstrap, 5000 iterations)						
Total effect: TS → Burnout	−0.05	0.06	−0.78	0.437	—	—
Indirect effect: TS → POS → Burnout	−0.19	0.06	—	0.002	−0.30	−0.08

Note. TS = technostress total composite; B = unstandardised path coefficient; SE = standard error; CI = bias-corrected bootstrap confidence interval; LL = lower limit; UL = upper limit. t-values for total and indirect effects not computed by bootstrap estimation. N = 150.

## Data Availability

Available upon request.

## Public Involvement Statement

No public involvement in any aspect of this research.

## Guidelines and Standards Statement

This manuscript was prepared in accordance with the STROBE (Strengthening the Reporting of Observational Studies in Epidemiology) guidelines for cross-sectional studies.

## Abbreviations

AGFI	Adjusted Goodness of Fit Index
CBI	Copenhagen Burnout Inventory
CFI	Comparative Fit Index
CI	Confidence Interval
COR	Conservation of Resources Theory
EHR	Electronic Health Record
GFI	Goodness of Fit Index
ICT	Information and Communication Technologies
IRB	Institutional Review Board
JD-R	Job Demands–Resources Model
NFI	Normed Fit Index
POS	Perceived Organizational Support
RMSEA	Root Mean Square Error of Approximation
SD	Standard Deviation
SE	Standard Error
SEM	Structural Equation Modelling
TLI	Tucker–Lewis Index
TS	Technostress (Total Composite Score)
VIF	Variance Inflation Factor
